# Viral Agents Causing Acute Respiratory Infections in Children under Five: A Study from Eastern India

**DOI:** 10.1155/2016/7235482

**Published:** 2016-11-27

**Authors:** Pravakar Mishra, Lipika Nayak, Rashmi Ranjan Das, Bhagirathi Dwibedi, Amitabh Singh

**Affiliations:** ^1^SVP Post Graduate Institute of Paediatrics, SCB Medical College & Hospital, Cuttack 753007, India; ^2^Department of Pediatrics, All India Institute of Medical Sciences, Bhubaneswar 751019, India; ^3^Regional Medical Research Centre (ICMR), Bhubaneswar 751016, India; ^4^Department of Pediatrics, Chacha Nehru Bal Chikitsalaya, New Delhi 110031, India

## Abstract

*Background*. Acute respiratory infections (ARIs) are important cause of mortality and morbidity in children under five in developing country.* Methods*. This observational study was conducted over two-year period in a tertiary care teaching hospital of Eastern India. Nasal and throat swabs were collected, transported to the laboratory at 2–8°C in viral transport media, and then processed for detection of viruses using mono/multiplex real-time polymerase chain reaction.* Results*. A total of 300 children aged 2–60 months with ARIs were included. The most common age group affected with LRI was 2–12 mo and with URI was >12–60 mo. Viruses were detected in 248 cases. In URI, 77 were positive for single virus and 19 were positive for more than one virus; in LRI, 113 were positive for single virus and 12 were positive for more than one virus. The most common viruses isolated from URI cases were rhinovirus and adenovirus. The most common viruses isolated from LRI cases were respiratory syncytial virus and influenza virus. Most cases occurred in the months of January, December, and August.* Conclusion*. Viruses constitute a significant cause of ARI in children under five. RSV, ADV, RV, and IFV were the most prevalent viruses isolated.

## 1. Background

Acute respiratory infections (ARIs) significantly impact the health of children worldwide. Though the pathogens causing ARIs vary geographically and by season, globally viruses play a major role [1–3]. In a recent systematic review, the most common respiratory viruses causing acute lower respiratory tract infection (LRI) in children under five years of age were respiratory syncytial virus (RSV), influenza virus (IFV), parainfluenza virus (PIV) human metapneumovirus (hMPV), and rhinovirus (RV) [1]. Besides this, 10%–50% of children affected with ARI develop secondary bacterial infections, namely, acute otitis media, sinusitis, or pneumonia [4]. Moreover, viruses are the most common pathogen associated with severe respiratory diseases (e.g., bronchiolitis), exacerbation of asthma, or pneumonia in early life and are leading cause of hospitalization in children under two [5–7]. Although viral etiology of ARIs and their impact on health care are much studied in developed countries, there is a gap in knowledge regarding the same in developing countries including India [8]. From the public health point of view, it is important to know the most common viral agents causing ARIs, their manifestations, how often they cause severe disease, and how severe ARIs can be prevented. In this study, we aimed to characterize the viral spectrum and pattern of upper and lower ARIs in children under five from eastern part of India.

## 2. Methods

The present study was conducted in the pediatrics department of SVPPGIP, SCB Medical College, a tertiary care teaching hospital in Eastern India over 2-year period (October 2011 to September 2013). Children aged 2 to 60 months with symptoms and signs of acute respiratory tract infections suggested by WHO were included [9]. WHO classification of acute respiratory tract infection in children presenting with cough, difficult breathing, or both is as follows: pneumonia—respiratory rate per minute >50 breaths (2–11 months of age) or >40 breaths (12–59 months of age); no lower chest indrawing; severe pneumonia—symptoms of pneumonia, and lower chest indrawing with or without rapid breathing; very severe disease—symptoms of severe pneumonia, inability to drink, convulsions, central cyanosis, being abnormally sleepy or difficulty to wake, stridor in calm child, or clinically severe malnutrition. Those with suspected bacterial etiology (e.g., streptococcal sore throat, lobar pneumonia, pneumatocele, and empyema), underlying chronic conditions, HIV, illness lasting more than a week, severe malnutrition, and those hospitalized in last one month were excluded. The Institute Ethics Committee approved the study. Written informed consent was obtained from the parents/legal guardians.

Nasal and throat swabs were collected and pooled into tubes containing 2 mL of virus transport medium (VTM, Copan, Brescia, Italy) and kept in refrigerator at 2 to 8°C. Then the samples were transported over ice to the laboratory (Regional Medical Research Centre, ICMR) situated at Bhubaneswar, a Grade 1 equipped virological laboratory [10]. Samples were kept there in −80°C until further processing. Total nucleic acids (including DNA and RNA) were extracted from 200 *μ*L of each specimen using a QIAamp MinElute Virus Spin Kit (Qiagen, Mississauga, ON, Canada) according to the manufacturer's instructions. For all collected specimens, PCR or RT-PCRs were performed to detect infection with RSV, IFV, PIV, hMPV, adenovirus (ADV), human bocavirus (hBoV), human corona virus (hCoV), enterovirus (EV), and RV. The method used for each virus is described in the following references: PIVs 1–4, EV, RV [8], IFV (A, B, and C), and RSV (A and B) [11] were detected by two multiplex nested RT-PCRs; hCoV [12] and hMPV [13] by two-step RT-PCR; ADV [14] by single-step PCR; and hBoV by touchdown PCR [15]. RT-PCR was performed using a SuperScript II one-step RT-PCR Platinum Taq kit (Invitrogen, Carlsbad, CA, USA). PCR was performed using ExTaq polymerase (Takara, Otsu, Japan). All products underwent electrophoresis in 2% agarose gel. Typing for IFV, PIV, and RSV was performed according to PCR product size.

## 3. Results

A total of 358 children of 2–60 mo age and acute respiratory infections were screened and finally 300 patients were included after meeting the eligibility criteria. Following were the reasons for exclusion in 58 children: chest X-ray suggestive of bacterial pneumonia (*n* = 19), streptococcal sore throat (*n* = 15), underlying chronic conditions (*n* = 10), severe malnutrition (*n* = 7), illness lasting more than a week (*n* = 3), complicated pneumonia (*n* = 2), and being hospitalized in last one month (*n* = 2). The M : F ratio was 1.7 : 1 (URI = 1.6 : 1; LRI = 1.9 : 1). Out of 300, 174 (58%) were LRI and 126 (42%) were URI cases.

**Figure 1:**
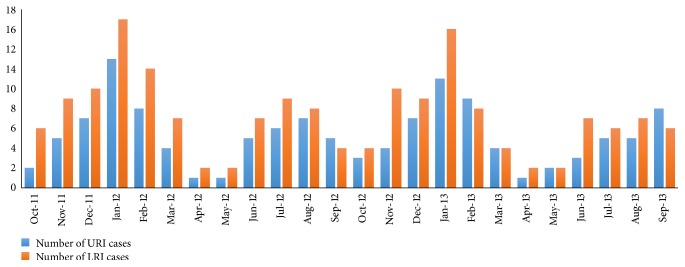
Month-wise recruitment of URI and LRI cases.

Month-wise recruitment of URI and LRI cases has been shown in Figure 1. Most URI cases occurred in the month of January and December. Most LRI cases occurred in the month of January, August, November, March, and July. Most children with LRI were in the age range of 2–12 mo (54%) and with URI were in the age range of >12–60 mo (45.2%). Auscultatory wheeze was present in 85% and crackles in 82% of LRI cases. Chest X-ray findings were hyperinflation (93%), peribronchial cuffing (60%), interstitial infiltrates (48%), and patchy opacity (46%).

**Figure 2:**
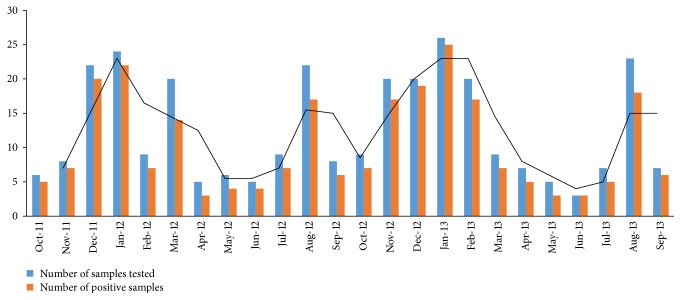
Month-wise recruitment of ARI cases and samples positive for viruses.

Of the 300 nasal and throat swabs, 248 cases were positive for viruses [LRI = 142/174 (81.6%), and URI = 106/126 (84.1%)]. Month-wise recruitment of ARI cases and samples positive for viruses has been shown in Figure 2. The maximum number of cases recruited and samples positive for viruses were in the months of January, December, and August. The respiratory viruses found in URIs and LRIs are shown in Table 1. The virus positivity in ARI was as follows: URI, 65 (61.3%) samples were positive for single virus and 41 (38.7%) were positive for more than one virus, and LRI, 86 (61%) samples were positive for single virus and 56 (39%) were positive for more than one virus. The coinfection pattern differed between acute URI and LRI, and this has been detailed in Table 2. The most common viruses isolated from URI cases were RV (31.1%), ADV (18.9%), RSV (17%), and IFV (17%). The most common viruses isolated from LRI cases were RSV (30.3%), IFV (17.6%), RV (14.8%), and ADV (13.4%). RSV, ADV, RV, and IFV were the most prevalent viruses isolated from all the ARI cases. IFV-A was predominant over IFV-B, and PIV-3 was predominant over other subtypes of PIV. RSV-A subtype dominated over RSV-B in URI cases, whereas RSV-B dominated over RSV-A in LRI cases. PIV-2 and 4, hCoV, and EV were not detected in URI cases.

The detection rates of viruses corresponding to different age groups are shown in Table 1. In case of URIs, RV was the most prevalent virus (2–6 mo age = 2.8%; >6–12 mo age = 13.3%; >12–60 mo age = 14.2%). RSV was the second most common virus detected in age groups of 2–6 mo and >6–12 mo. ADV was the second most common virus detected in age group of >12–60 mo. hMPV and hBoV were not detected up to 12 mo age. The least common viruses in all age groups were PIV, hBoV, and hMPV. hCoV and EV were not detected in any age group. In case of LRIs, RSV was the most prevalent virus (2–6 mo age = 13.4%; >6–12 mo age = 11.3%; >12–60 mo age = 5.6%). IFV was the second most common virus detected in all age groups. hMPV, hBoV, and hCoV were not detected up to 6 mo age. EV was not detected up to 12 mo age. The least common viruses in all age groups were hBoV, hCoV, EV, and hMPV.

**Figure 3:**
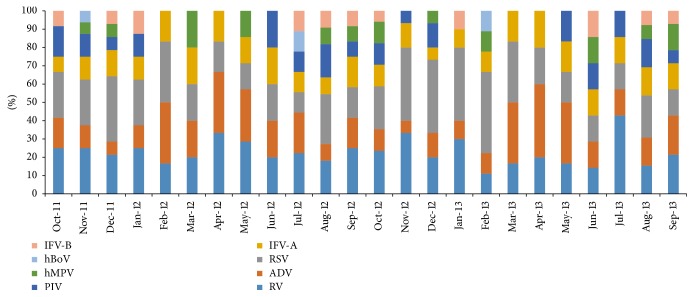
The distribution of viruses by months of recruitment.

The distribution of viruses by month has been shown in Figure 3. There was difference between URIs and LRIs. RV was the most common virus detected in URIs, but it was fourth most prevalent in LRIs. It was detected throughout the year, the maximum rate being in December, January, March, and August. RSV was the most common virus detected in LRIs, but it was third most prevalent in URIs. It was detected throughout the year, the maximum rate being in December, January, and February. IFV was second most common virus detected in LRIs, but it was third most prevalent in URIs; maximum positivity rate was in August followed by in July, and minimum positivity rate was in November. The trend for PIV differed from that of IFV. PIV had a higher prevalence in April to July, and a low prevalence from September to March. EV and hCoV 43 were not detected in URIs. There detection rate was lower in LRIs. Like hMPV, hBoV was present throughout the year in LRIs.

The clinical features, demographics, and risk factors of children among viral positive and virus negative group with LRIs were compared (Table 3). It was observed that significantly higher number of children below 12 mo were virus positive (*p* = 0.01). Children presenting with preceding bronchiolitis were significantly associated with total viral infections (*p* = 0.003). Rhinorrhea was significantly present in the virus positive group (*p* = 0.02). Among risk factors, ARI in family was significantly associated with virus positivity (*p* = 0.04).

In LRIs, out of 174 cases (LRI = 101; severe LRI = 51; very severe LRI = 22), 161 were cured and 13 died. Of 13 cases who died, 10 had coinfection with more than 1 respiratory virus (IFV + RSV = 5; RSV + RV = 3; IFV + hCoV43 + RV = 2). In URIs, all were cured.

## 4. Discussion

In the present study, respiratory viruses were detected in 248 cases (LRI = 142; URI = 106). RSV (25.8%), RV (21.9%), IFV (21%), and ADV (12.5%) were the most prevalent single viruses isolated from all the ARI cases. The most common viruses isolated from acute URI cases were RV (32.6%), ADV (19.6%), RSV (17.4%), and IFV-A (15.2). The most common viruses isolated from acute LRI cases were RSV (32.4%), IFV-A (19.6%), ADV (18.6%), and RV (15.7%). Most cases occurred in the month of January, December, and August. In URIs, the most common age group affected was >12–60 mo, and in LRIs, the most common age group affected was 2–12 mo.

Present study results are similar to the previous study results from India. In the study by Broor et al., RSV, IFV-A, and PIV-3 were important causes of ARI in children under five in the rural Indian community [8]. In the study by Singh et al., RSV was the most frequently detected virus (21.3%) from hospitalised children presenting as ALRI followed by IFV (9.0%), measles (8.5%), and ADV (5.3%) [16]. In the study by Bharaj et al., RSV was the most frequently detected virus (58.1%) from hospitalised children presenting as ALRI followed by PIV (22%), hMPV (10.5%), and IFV-A (9.3%) [17]. In the study by Maitreyi et al., RSV was the most frequently detected virus (17%) from hospitalised children presenting as ALRI followed by IFV (14.5%), PIV (11.5%), and ADV (1.5%) [18]. In the study by Yeolekar et al., RSV was the most frequently detected virus (26%) from hospitalised children presenting as ALRI followed by IFV (5.4%), PIV (2.07%), and ADV (0.8%) [19]. Panda et al. recently found RV as the most frequently detected pathogen (24.7%) followed by RSV (4.22%), PIV (2.11%), and hMPV (2.11%) in children with ARI from Eastern India [20]. In the present study, we found RV as the second most important agent to RSV causing ARI in young infants and children. The present study findings are more or less similar to the recently published studies from other parts of the globe, though the most prevalent virus may be different [21–27].

The viral URI and LRI prevalence varied with age, URIs being common in children of >12–60 mo and LRIs being common in infants of <12 mo age. Similar was finding by previous studies [8, 16, 17]. Infants <12 mo age were most commonly positive for RSV and less commonly for IFV, the latter occurring commonly beyond infancy in older children. This is because maternal antibody protection against RSV during infancy remains questionable, and as the child grows, the immunity builds up against RSV due to repeated exposures [28]. IFV due to marked antigenic diversity evades the herd immunity and does not give long lasting immunity. Therefore, the susceptibility to IFV increases as the child grows [29]. ADV is uncommon during first six months, where maternal antibody confers protection [30].

Previous Indian studies [16, 17] have compared the clinical, demographic, and risk factors for ALRI testing positive for respiratory viruses with those testing negative for respiratory viruses. One study observed that significantly higher number of children below 12 mo of age had RSV infection, those presenting with preceding bronchiolitis were significantly associated with virus positivity, and ARI in family was significantly associated with virus positivity [17]. In another study, patients testing positive for viruses showed significantly high percentage of cough, nasal discharge, and diarrhea [16]. We found a significantly higher number of children below 12 mo being virus positive, those presenting with preceding bronchiolitis being significantly associated with virus positivity, rhinorrhea significantly presenting in the virus positive group, and ARI in family being significantly associated with virus positivity.

The respiratory illness exhibits a clear-cut seasonality, and all the published studies till date support this. However, there may be some difference in the seasonality. Most of the studies report increased incidence of acute respiratory infection (both URI and LRI) during winter and fall season [20–27]. This is because of the following sequence of events: decrease in air temperature causing decrease in the nasal airway temperature (compromised cooling of the nasal airway) leading to poor defence against infection (because of decrease mucociliary clearance and phagocytosis) [31]. In the present study, the incidence of both URI and LRI was higher in winter season (as upper airway acts as an entry point to lower airway). However, an additional peak of acute LRI was also found during August. Though this cannot be solely explained based on the above hypothesis, it may be because of an increase in the birth cohort during this period [32].

In acute LRIs, severe clinical phenotypes (severe and very severe pneumonia, acute respiratory distress syndrome) were more prevalent in case of coinfection with more than one respiratory viruses. Of 13 patients who died, 10 were because of coinfection. Similar findings have been observed by authors from India and abroad [20–27].

The present study findings throw lights on the management of an individual case as well as the community. First, good hand hygiene, maintenance of warm temperature in the living place/avoidance of exposure to sudden cold temperature, avoidance of taking of freeze dried/cold food items, and wearing of the mask to prevent spread of the infection should be practiced. Second, the nutritional status of the children in the community should be improved as malnutrition predisposes to increase risk of infection and severe illness. Third, vaccines should be developed against respiratory viruses (including against the new viruses) to decrease the morbidity and mortality from respiratory illness. Fourth, any child with ARI and viral coinfection should be closely monitored for development of complications/severe illness.

Strengths of present study are adequate sample size and children being recruited over 2 consecutive years including all the seasons. Limitations include children presenting to the health facility only were recruited, reinfection rate by the viruses were not considered, and simultaneous/secondary bacterial infections were not studied.

## 5. Conclusion

RSV, ADV, RV, and IFV were the most prevalent viruses isolated from the ARI cases. The epidemiology of respiratory viruses needs to be studied further for vaccine development and implementation purpose. Any child with ARI and viral coinfection should be monitored for development of complications/severe illness.

## Figures and Tables

**Table 1:** Isolation of viruses (monoinfection versus coinfection) in children with ARIs.

Viruses	Total	Monoinfection			Coinfection		
		2–6 mo	>6–12 mo	>12–60 mo	2–6 mo	>6–12 mo	>12–60 mo
Acute URI (percentage) (*N* = 106)							
RSV	18 (17)	3 (2.8)	7 (6.6)	4 (3.8)	0	1 (0.9)	3 (2.8)
RSVA	13 (12.3)	2 (1.9)	5 (4.7)	3 (2.8)	0	1 (0.9)	2 (1.9)
RSVB	5 (4.7)	1 (0.9)	2 (1.9)	1 (0.9)	0	0	1 (0.9)
ADV	20 (18.9)	0	2 (1.9)	7 (6.6)	1 (0.9)	2 (1.9)	8 (7.6)
RV	33 (31.1)	3 (2.8)	8 (7.6)	11 (10.4)	1 (0.9)	6 (5.7)	4 (3.8)
IFV	18 (17)	1 (0.9)	4 (3.8)	6 (5.7)	0	3 (2.8)	4 (3.8)
IFVA	15 (14.2)	1 (0.9)	3 (2.8)	5 (4.7)	0	3 (2.8)	3 (2.8)
IFVB	3 (2.8)	0	1 (0.9)	1 (0.9)	0	0	1 (0.9)
hMPV	6 (5.7)	0	1 (0.9)	3 (2.8)	0	0	2 (1.9)
hBoV	4 (3.8)	0	0	2 (1.9)	0	1 (0.9)	1 (0.9)
PIV	7 (6.6)	0	1 (0.9)	2 (1.9)	0	2 (1.9)	2 (1.9)
PIV1	2 (1.9)	0	0	0 (0.9)	0	1 (0.9)	1 (0.9)
PIV2	0	0	0	0	0	0	0
PIV3	5 (4.7)	0	1 (0.9)	2 (0.9)	0	1 (0.9)	1 (0.9)
PIV4	0	0	0	0	0	0	0
hCoV 43	0	0	0	0	0	0	0
EV	0	0	0	0	0	0	0

Acute LRI (percentage) (*N* = 142)							
RSV	43 (30.3)	16 (11.3)	9 (6.3)	3 (2.1)	3 (2.1)	7 (5.0)	5 (3.5)
RSVA	17 (12)	4 (2.8)	5 (3.5)	1 (0.7)	2 (1.4)	4 (2.8)	1 (0.7)
RSVB	26 (18.3)	12 (8.5)	4 (2.8)	2 (1.4)	1 (0.7)	3 (2.1)	4 (2.8)
ADV	19 (13.4)	1 (0.7)	3 (2.1)	7 (5.0)	0	3 (2.1)	5 (3.5)
RV	21 (14.8)	2 (1.4)	5 (3.5)	7 (5.0)	1 (0.7)	2 (1.4)	4 (2.8)
IFV	25 (17.6)	2 (1.4)	5 (3.5)	8 (5.6)	1 (0.7)	4 (2.8)	5 (3.5)
IFVA	17 (12)	2 (1.4)	4 (2.8)	6 (4.2)	1 (0.7)	2 (1.4)	2 (1.4)
IFVB	8 (5.6)	0	1 (0.7)	2 (1.4)	0	2 (1.4)	3 (2.1)
hMPV	8 (5.6)	0	1 (0.7)	3 (2.1)	0	2 (1.4)	2 (1.4)
hBoV	7 (4.9)	0	1 (0.7)	2 (1.4)	0	1 (0.7)	3 (2.1)
PIV	15 (10.5)	1 (0.7)	4 (2.8)	4 (2.8)	1 (0.7)	3 (2.1)	2 (1.4)
PIV1	5 (3.5)	0	1 (0.7)	1 (0.7)	0	1 (0.7)	2 (1.4)
PIV2	1 (0.7)	0	0	1 (0.7)	0	0	0
PIV3	7 (4.9)	1 (0.7)	2 (1.4)	2 (1.4)	1 (0.7)	1 (0.7)	0
PIV4	2 (1.4)	0	1 (0.7)	0	0	1 (0.7)	0
hCoV 43	3 (2.1)	0	1 (0.7)	1 (0.7)	0	0	1 (0.7)
EV	1 (0.7)	0	0	0	0	0	1 (0.7)

RSV: respiratory syncytial virus; ADV: adenovirus; RV: rhinovirus; IFV: influenza virus; hMPV: human metapneumovirus; hBoV: human bocavirus; PIV: parainfluenza virus; hCoV 43: human corona virus 43; EV: enterovirus; ARI: acute respiratory infection; URI: upper respiratory infection; LRI: lower respiratory infection.

**(a):** 

Acute URI coinfection types	Number
RV + RSV	9
RV + ADV	7
IFV + PIV	6
RV + IFV	4
PIV + RV	5
RSV + hBoV	3
RSV + hMPV	2
RV + hMPV + hBoV	3
RV + ADV + RSV	1
PIV + ADV + IFV	1

*Total *	*41*

**(b):** 

Acute LRI coinfection types	Number
IFV + RV	10
ADV + IFV	9
RV + IFV	8
RV + RSV	12
IFV + RSV	7
IFV + EV	1
RV + hMPV	1
PIV + ADV	1
RV + hMPV + ADV	1
IFV + hCoV43 + RSV	2
RSV + hMPV + ADV	2
IFV + hCoV43 + RV	2

*Total*	*56*

**Table 3:** Clinical and demographic profile of patients with ALRI as per virus isolation status.

Variables	Virus positive (*n* = 142)	Virus negative (*n* = 32)	OR (95%CI)	*p* value
Age (median)	11 mo	14 mo	—	0.42
Male : female	1.84 : 1	1.46 : 1	1.21 (0.54–2.62)	0.67
<12 mo	88	12	2.7 (1.24– 6.1)	0.01^*∗*^
Risk factors				
ARI in family	77	11	2.24 (1.06–5.12)	0.04^*∗*^
Prematurity	54	08	1.86 (0.74–4.41)	0.16
Smokers in house	69	12	1.54 (0.68–3.48)	0.25
Use of cooking fuel	81	15	1.48 (0.64–3.32)	0.32
Symptoms				
Cough	140	31	2.26 (0.18–25.7)	0.5
Fever	122	24	2.04 (0.76–5.24)	0.13
Rhinorrhea	105	17	2.5 (1.14–5.58)	0.02^*∗*^
Sore throat	047	09	1.28 (0.53–2.95)	0.58
Breathing difficulty	075	12	1.83 (0.84–4.11)	0.12
Febrile convulsion	007	01	1.6 (0.18–13.56)	0.66
Signs				
Grunting	19	03	1.47 (0.42–5.36)	0.54
Nasal flaring	27	04	1.64 (0.52–5.04)	0.39
Chest indrawing	57	08	2.01 (0.82–4.76)	0.11
Stridor	02	0	—	0.48
Cyanosis	02	01	0.44 (0.02–5.1)	0.5
Clinical diagnosis				
Bronchiolitis	91	11	3.21 (1.42–7.35)	0.003^*∗*^
Pneumonia	76	18	0.9 (0.38–1.98)	0.78
Myocarditis	02	0	—	0.48

^*∗*^ *p* value < 0.05; ALRI: acute lower respiratory infection; OR: Odds ratio; 95% CI: 95% confidence interval.
